# Corpus-based critical discourse analysis of reporting practices in English news reports on public health event in China and United States

**DOI:** 10.3389/fpsyg.2023.1137382

**Published:** 2023-03-08

**Authors:** Zhuoran Li, Ronghui Zhao, Baocui Lou

**Affiliations:** ^1^Institute of Linguistics, Shanghai International Studies University, Shanghai, China; ^2^Faculty of International Studies, Henan Normal University, Xinxiang, China

**Keywords:** reporting practice, reporting verbs, news reports, critical discourse analysis, corpus

## Abstract

**Introduction:**

Reporting speech is a basic form of human language, and reporting practices play a key role in news report. As one of the important rhetorical devices to introduce the reported speech, reporting verbs can help the readers understand the source of the reported speech and the attitude of the journalist or the media toward the reported information.

**Method:**

This study examines the features of reporting practice in Chinese and American news reports on public health emergency by investigating the use of reporting verbs from the perspectives of critical discourse analysis. Two English news corpora of COVID-19 pandemic are built, namely, the China Daily News Corpus and the New York Times News Corpus, with 50 news texts in each corpus. The corpus analysis tool AntConc 3.3.5 is used to conduct concordance analysis.

**Results and discussion:**

It is found that Chinese and American news reports tend to use roughly the same high-frequency reporting verbs in reporting the COVID-19 pandemic. Chinese and American news corpora show difference in the distribution feature of high-frequency reporting verbs in terms of semantic category. Both Chinese and American news reports use speech reporting verbs most frequently, indicating an objective attitude toward the reported event, and use speech reporting verbs and speech act reporting verbs to introduce the reported speech with comparatively higher degree of certainty. American news reports frequently use mental reporting verbs to show the attitude of uncertainty toward the reported speech, and Chinese news reports probably need to raise the awareness of using mental reporting verbs to express the opinions and attitude of the common people or the authority. The findings of this study can provide insights into the research on reporting strategies of news reports on emergencies in China for foreign audience.

## 1. Introduction

Reporting speech is a basic form of human language, and reporting practices play a key role in news report. As one of the important rhetorical devices to introduce the reported speech, reporting verbs can help the readers understand the source of the reported speech and the attitude of the journalist or the media toward the reported information. In a public health emergency, people tend to search frequently for information related to their health and safety due to worries about unknown future events (McMullan et al., 2019; Liu et al., 2021). News report serves as a reliable source of information by showing the recently-occurring fact, and thus is always taken as the main source for information acquisition. Being a critical lexical device to introduce others’ speech, the reporting verbs contribute to intensifying the reliability of the information, and have therefore attracted much attention in the study of reporting practice in news texts.

News report is seen as the outcome of social practice, and has become a research interest in the field of discourse analysis. News media is the main channel for the government’s foreign-oriented publicity and is used to spread real events to the public, which can satisfy the public’s need to know about what is happening and guide the trend of public opinion at the same time (Guo, 2011). The main task of the news press is information processing. Emergencies are public and harmful, so news press should play the role of communicating with the public, society and government, guide and supervise the trend of public opinion properly (He, 2008). News reports on serious topics are supposed to impartially present the reported events to readers, provide the voices of various sources of information, and rarely use words that express personal feelings or attitudes. In international communication, the choice of information resources and report strategies in news texts will affect the construction of the national identity and the construction of the international communication discourse system.

Language is usually not considered to be a neutral medium of communication, and it does not simply reflect the reality of social activity, but is a constructive mediator that helps to construct the ideas and beliefs that powerful classes need to spread (Fairclough, 1989; Fowler, 1991). News reports are supposed to present the actual event, provide different voices from various sources, and exclude the personal opinion and ideological intervention of the journalist or the media. The journalist is believed to describe the real events that have actually happened in an objective manner without taking into consideration his or her own attitude toward the reported event (Li, 2008; Kang and Li, 2018). In reality, news discourse often provides us with more than two voices through reporting practice. The importance of reporting practice in news discourse is not only to provide readers with information from different sources, but also to show the attitude and ideological tendency of the media (Chen, 2010; Kang and Li, 2018). The choice of reported speech can demonstrate the journalist’s ideology and value standpoint (Yang and Xin, 2021). That is to say, whose words are quoted, which words are quoted, and how they are chosen will reflect the attitude and stance of the journalist and the news media.

Reporting practices are found to be frequently used in the news discourse (Biber et al., 1999) because news is much more about what people say than what is done (Floyd, 2000) and that reporting information from different sources is a common practice in journalism. Previous studies on reporting practices are mainly conducted from the perspectives of reporting verbs, reporting mode (e.g., direct/indirect speech), reported content, and source of the reported speech (Chen, 2010). Reporting verbs are most important and frequently used linguistic signals in reporting practices to introduce others’ speech or ideas (Hyland, 2002; Chen, 2010).

Reporting verbs are used to convey the words or opinions of others, constitute the most direct context of reported speech, and can express the journalist’s interpretation of the details of the reported speech (Xin and Gao, 2019). Recent research has shown that the choice of reporting verbs can indicate their attitude or stance toward the current proposition by different linguistic choices (Chen, 2010; Kang and Li, 2018; Xin and Gao, 2019) and that news reports, though apparently neutral, can show personal preferences of the journalists by using different reporting verbs (Floyd, 2000; Xin, 2008). The choice of specific reporting verbs can reflect the journalist’s attitude toward the reported information and affect the judgment of the reader toward the reported information (Floyd, 2000; Chen, 2010; Kang and Li, 2018).

Studies on reporting verbs are mainly conducted in two genres: the news discourse and the academic discourse. The classification of reporting verbs is different due to the difference in the purpose of these two genres. Thompson (1996) argues that choice of reporting verbs can indicate the journalist’s attitude toward the truth of the reported message and the speaker, and classifies reporting verbs into three types in terms of the pragmatic function, namely neutral, positive, and negative. Based on the contrastive analysis of Japanese and American newspaper reports, Yamashita (1998) classifies reporting verbs into four types: reporting verbs (e.g., *say, tell*), mental (thought) verbs (*wonder, think*), manner of speaking verbs (*shout, howl*), and speech act (illocutionary) verbs (e.g., *warn, confirm*). In the study on academic discourse, Hyland (2002) classifies reporting verbs into three types in terms of the process function, namely, the research acts (real-world) verbs (e.g., *notice, show*), cognition acts verbs (*believe, think*), and discourse acts verbs (*confirm, report*).

In their comparative study of reported speech in Chinese and American newspapers, by referring to previous related studies (especially Yimashita’s study), Xin and Gao (2019) propose that reporting verbs can be divided into three types in terms of semantic category: speech reporting verbs, mental (thought) reporting verbs, and speech act reporting verbs. Speech reporting verbs are the most frequently used reporting verbs in news reports, aiming to present the reporting discourse objectively and as neutrally as possible, for example, *say* and *tell*. They can therefore increase the credibility of the report and reduce the intervention of the journalist or the media. Mental reporting verbs are used to express the thoughts and mental activities of the person being reported, and occur relatively rarely in news reports, for example, *believe* and *think*. Speech act reporting verbs are used to express the journalist’s judgment of the manner of reporting practice by emphasizing the way that the message is released by the speaker, for example, *announce*, and *confirm*. They can affect the readers’ understanding of the reported message. Xin and Gao’s classification (2019) is based on a comprehensive study of reporting verbs in 200 English news texts, with 7,310 occurrences and 279 types of reporting verbs, and is clear and practical in analyzing reporting verbs in news texts by listing the verbs of each type in an exhaustive way. This study will therefore adopt their classification targeted at the genre of news reports.

Previous studies on reporting verbs were mostly conducted in the genre of academic English (e.g., Hyland, 2002; Lou, 2011; Liardét and Black, 2019), and few studies have been conducted in news discourse from the perspectives of critical discourse analysis. By adopting the corpus-based approach, this study examines the features of reporting practices in Chinese and American news reports on public health emergency, the COVID-19 pandemic, by investigating the features of reporting verbs used in *China Daily* and *New York Times*, and analyzes the rhetorical strategy of Chinese media in international communication. Specifically, this study will address the following research questions: (1) What are the high-frequency reporting verbs used in Chinese and American news reports on the COVID-19 pandemic?, (2) What is the distribution feature of these high-frequency reporting verbs in terms of semantic category in Chinese and American news corpora? and (3) What are the similarities and differences in the reporting strategies used in Chinese and American news reports on the pandemic?

## 2. Materials and methods

### 2.1. Corpus-based approach

Corpus is generally defined as a collection of naturally-occurring language texts that has been built for a particular purpose (Sinclair, 1991). Through the study of authentic texts, corpus-based approach can uncover characteristics of language that were previously unsuspected (Biber et al., 1999), and help people understand the nature of language in a new perspective (Qian, 2010). Researchers can use computer software to process and analyze language data, and help people discriminate patterns in language use.

The computer software enables researchers to obtain the information on the wordlist of a corpus, the concordance lines and collocates of given items, and the lexical bundles in a corpus. Corpus-based approach makes it possible for researchers to objectively observe regularities of given linguistic items in language use. The corpus-based analysis on given search items, lexical bundles and collocations can show the feature of qualitative analysis, which makes the findings more reliable and comprehensive. Applying corpus-based approach to discourse analysis can reduce researcher bias due to the naturalness of language data and the comparatively objective judgment based on a large amount of data (Baker, 2006). Therefore, corpus-based analysis is often combined with discourse analysis in analyzing language use in the description of social events.

Corpus-based approach is used in this study to obtain the wordlist data of the news texts, to retrieve the concordance lines of reporting verbs, to calculate the frequency of each reporting verb, and to access the linguistic context of reporting verbs for discourse analysis.

### 2.2. Critical discourse analysis

Critical Discourse Analysis (CDA) is defined as a form of discourse analysis that takes a critical stance toward how people use language (Richards and Schmidt, 2010). It analyzes texts and other discourse types in order to identify the ideology and values underlying them. Fairclough (1992) developed a three-dimensional framework for CDA --- analysis of discourse as text, as discourse practice (processes of text production, distribution and consumption), and as social practice in order to emphasize that text analysis is not something that should be done in isolation. This framework was further described as analysis of language texts, analysis of discourse practice and analysis of discursive events as instances of sociocultural practice (Fairclough, 2010). In this framework, each language use can be interpreted as a communication event consisting of text, discourse practice and social and cultural practice. Accordingly, the analysis should specify the linguistic features, process of production and consumption from the perspective of audience and wider social practice.

CDA can reveal the interests and power relations in any institutional and socio-historical context through analyzing the ways that language is used. It is generally regarded as a trans-disciplinary, text-analytical approach to critical social research, and centers on the systematic, text-based exploration of language to reveal its role in the workings of power and ideology in society by investigating the power imbalance in the use of language (Fairclough, 2010). This framework can create links between discourse study and sociology, and politics, and serves as a means of exploring the relationship between language and social-institutional practices, and the close links between language as discourse and broader social and political structures.

News reports are concerned with what is happening in the social life, which is closely related to social issues, and are therefore always the interest of CDA. Corpus-based study can withdraw the search items in an exhaustive way and analyze language use with the linguistic context of the given items. Studies have demonstrated that the corpora can play an important role in critical social research, and that the combination of CDA and corpus approach is proved to be an effective method to analyze news texts (Baker and McEnery, 2005; Qian, 2010; Tang, 2011; Wu, 2017; Xin and Gao, 2019; Zhao and Zhang, 2019).

In this study, the three-dimensional framework proposed by Fairclough (1992) is used for further analysis based on the results of the corpus-based analysis. The “text” dimension is concerned with language analysis of the texts, to be more specific, the analysis of the linguistic features of reporting verbs, which is corpus-based analysis. The “discourse practice” dimension deals with the production and consumption of the news texts, and analyzes the reporting strategy employed by different news media based on the semantic features of high-frequency reporting verbs chosen in the news reports. The “social practice” dimension explains the relationship between the discourse practice and the social context by analyzing the social and cultural context of the reporting events.

### 2.3. Corpora building

Two English news corpora of COVID-19 pandemic are built, namely, the China Daily News Corpus (CDNC) and the New York Times News Corpus (NYTNC). With the *coronavirus, pandemic* and *virus* as the search word, 50 English news texts were chosen and downloaded, respectively, from the official website of *China Daily* and *New York Times*, and the time span of the news is from February to April 2020. The size of CDNC and NYTNC is 28,895 and 37,292, respectively, and the average text length of CDNC and NYTNC is 578 words and 746 words, respectively. Only the title and the main text of the news report are included in the corpora, and the pictures and the related illustrations of pictures were excluded.

### 2.4. Research instruments

The research instruments employed in this study are AntConc 3.3.5, and Loglikelihood and Chi-square Calculator 1.0. AntConc is a freeware corpus analysis tool for concordancing and text analysis. In this study, it is used to withdraw the concordance lines of the reporting verbs in the news corpora, to calculate the frequency of reporting verbs, to identify the most frequently used ones, and to provide the linguistic context of these reporting verbs.

Loglikelihood and Chi-square Calculator is used to calculate the *p* value of the observed data and decide whether there exists significant difference in the distribution of a given linguistic item or items between Chinese and American news reports.

### 2.5. Research procedure

The research procedure involved in this study includes:

1. Building the corpora. The corpora used in this study are two self-built corpora, the China Daily News Corpus and the New York Times News Corpus. In the process of corpora building, the theme (title), the number of texts, and the time span of the news are taken into consideration, and this makes the two corpora comparable.
2. Identifying and classifying reporting verbs. Reporting verbs to be examined are identified and classified based on the study of Hyland (2002) and Xin and Gao (2019). In their studies, nearly all the reporting verbs are identified. This study will take all the verbs listed as the search word to obtain the related frequency data.
3. Retrieving concordance lines and identifying the high-frequency reporting verbs. AntConc 3.3.5 is used to retrieve the concordance lines of the reporting verbs identified in step (2), including the –*ed* and *–s* forms of each item. In this process, the concordance lines in which the reporting verb is not used to report others’ speech or opinion are excluded, for example, the use of *report* as a noun. The data of each reporting verb is calculated, and the high-frequency reporting verbs can then be obtained.
4. Analyzing the semantic and stance feature of high-frequency reporting verbs. Based on the classification of Xin and Gao (2019), the high-frequency reporting verbs in each corpus are classified into three types, namely, speech reporting verbs, speech act reporting verbs and mental reporting verbs. The semantic and the stance feature of the high-frequency verbs in the two corpora are analyzed and compared from the perspectives of CDA.

## 3. Results

In this section, the top 10 high-frequency reporting verbs in each corpus will be identified, and the semantic category of these reporting verbs will be analyzed.

### 3.1. Results of the high-frequency reporting verbs in the two corpora

AntConc 3.3.5. is used to retrieve the concordance lines of each reporting verb and its inflectional forms, and then the top 10 high-frequency reporting verbs in the two corpora are obtained (see Table 1).

**Table 1:** High-frequency reporting verbs in the two corpora.

China daily news corpus			New York times news corpus		
Reporting verbs	Frequency	Normalized frequency (per 10,000 words)	Reporting verbs	Frequency	Normalized frequency (per 10,000 words)
Say	320	111	Say	400	107
Report	51	18	Call	42	11
Add	46	16	Announce	41	11
Call	30	10	Report	41	11
Confirm	27	9	Ask	32	9
Tell	25	9	Think	29	8
Announce	21	7	Add	26	7
Show	17	6	Tell	23	6
Ask	16	6	Believe	18	5
Order	14	5	Confirm	18	5

Table 1 presents the raw frequency and normalized frequency of the top 10 high-frequency reporting verbs in the two corpora in the order of frequency. Among the top 10 verbs, eight verbs occur in both corpora, namely, *say, report, add, call, tell, announce, ask,* and *confirm*. The verbs *show* and *order* occur only in the Chinese news corpus, and *think* and *believe* occur only in the American news corpus. This result indicates that, in international communication, Chinese and American news reports tend to choose the same high-frequency reporting verbs. The verb *say* occurs most frequently in both corpora, with its frequency accounting for more than half of the total frequency of the top-ten frequently occurring reporting verbs, indicating that both Chinese and American news reports are generally presented in an objective manner, because the use of *say* indicates that the journalist presents the reported speech in a most neutral manner without incorporating his or her own attitude toward the current issue.

### 3.2. Distribution of the semantic categories of the high-frequency reporting verbs

Table 2 indicates the semantic category and the distribution feature of the high-frequency reporting verbs, based on the categorization of Xin and Gao (2019). After the frequency of each type is obtained, Loglikelihood and Chi-square Calculator is used to calculate the *p* value of the frequency of each type. Results show that: (1) Among these high-frequency reporting verbs, all the three types of verbs, speech reporting verbs, mental reporting verbs and speech act reporting verbs occur in the American news corpus; the speech verbs and speech act verbs occur in the Chinese news corpus, and mental reporting verbs are not frequently used in Chinese news reports, (2) Speech reporting verbs are most frequently used in both corpora, occupying 73 percent and 74 percent in Chinese and American news reports respectively, (3) The two corpora do not show significant difference in the total frequencies in the frequency of these top 10 reporting verbs, and (4) The frequency of occurrences of speech act reporting verbs and mental reporting verbs shows significant differences in the two corpora.

**Table 2:** Semantic category of the high-frequency reporting verbs in the two corpora.

Semantic category	China daily news corpus		New York times news corpus		Log. value	*p* value
	Frequency (percentage)	Normalized frequency (per 10,000 words)	Frequency (percentage)	Normalized frequency (per 10,000 words)		
Speech reporting verbs	412 (73%)	143	496 (74%)	133	1.086	0.297
Speech act reporting verbs	155 (27%)	54	127 (19%)	34	14.504	0.000^*^
Mental reporting verbs	0	0	47 (7%)	13	−53.928	0.000^*^
Total	567 (100%)	196	670 (100%)	180	2.382	0.123

^*^ stands for significant difference; −stands for less frequent use.

## 4. Discussion

In this section, the use of speech reporting verbs, speech act reporting verbs, and mental reporting verbs will be discussed.

### 4.1. Speech reporting verbs

Speech reporting verbs *say, report, tell* and *ask* occur frequently in both corpora, as can be seen in Table 1. There are 412 occurrences (143 occurrences per 10,000 words) in Chinese news corpus and 496 occurrences (133 occurrences per 10,000 words) in American news corpus, and there is no significant difference in the frequency of these speech reporting verbs in the two corpora. The frequency of this type of reporting verbs is obviously much higher than the other two types. The use of these verbs shows that the related reporting practice is neutral and most objective. These speech reporting verbs give no indication of the journalist’s attitude toward what is being reported (Thompson, 1996). The use of these verbs shows that news reports always emphasize the neutral position in the news event, and that personal feelings and attitude should not be involved in the report of the event. Journalists are supposed to hold an objective, rational, and impartial attitude toward the event, and therefore the readers should not be influenced by the journalist.

Both Chinese and American news reports frequently use this type of reporting verbs to report the current event in an objective and neutral manner, which indicates the feature of the news genre. The verb *say* occurs most frequently in both corpora to report others’ speech or opinion. For example,

1. Xu Nanping, vice-minister for science and technology, said in a news conference that Chinese scientists are researching multiple types of vaccines simultaneously, and vaccine development in China is on par with rest of the world. (CDNC)
2. Crucial signs demonstrating the effectiveness of China’s fight against COVID-19 are well in view. Last Thursday, China reported no local new cases of the novel virus, with serious cases decreasing by 173. (CDNC)
3. At a news conference on Saturday, the Science and Technology Department of Zhejiang Province **s**aid that their newly developed vaccine for the novel coronavirus had already produced antibodies and was now being tested on animals. (CDNC)
4. The governor **s**aid no gatherings of 500 or more people will be permitted until further notice. (NYTNC)

In these four sentences, the subjects of *said* or *report* are either the authority or the spokesman of an organization. This shows that that the reporting speech is objectively and timely conveyed to the public, which helps the readers or the public know more about the issue, attach importance to the issue and become convinced of the information issued. The speech reporting verbs are employed here to disseminate measures taken by the government to cope with the situation, to make known the casualty figures caused by the pandemic, or to reveal the improvement in vaccine research and development. The use of speech reporting verbs indicates that the journalist gives a neutral evaluation of reported information and that the reported information is not controlled by the journalist and is presented to the reader objectively (Yamashita, 1998).

In the early stage of this public health emergency, the public knows little about the virus, and is urgent to understand more about the nature of the virus, to know that the pandemic is under control, and to know that the government is managing to fight against the unknown virus by taking measures and developing vaccines. In the process of news production, the duty of the media is to reveal scientific knowledge of the disease, and to spread authoritative information on public protection measures in order to relieve the tension caused by the disease (Zheng and Zhao, 2020), which is realized by using these speech reporting verbs to introduce official and authoritative information in an objective manner. The content of the reported speech can meet the needs of common people and alleviate fear of the public.

### 4.2. Speech act reporting verbs

Speech act reporting verbs, *confirm, add, call, announce, show,* and *order* occur frequently in Chinese news report with 155 occurrences (54 occurrences per 10,000 words); speech act verbs *call，announce, confirm,* and *add* occur frequently in American news report with 127 occurrences (34 occurrences per 10,000 words). This type of verbs occurs more frequently in Chinese news reports partly due to the absence of the mental reporting verbs in the high-frequency verbs. These verbs can not only perform the function of reporting others’ speech, but also imply that the journalist thinks that the reported information is of higher degree of certainty. For example,

5. On Wednesday, the government announced it would ban entertainment venues such as mahjong parlors, karaoke lounges and nightclubs for 14 days in an urgent move to minimize social gatherings, after fitness centers, cinemas, and billiard and game centers were banned last week. (CDNC)
6. Yet Zhejiang’s breakthroughs are still innovative in two aspects. First, they have confirmed that S-protein of the novel coronavirus plays a key role in its intrusion into human cells, and the vaccine is specifically targeted at preventing this. (CDNC)
7. In a letter to students and faculty at Columbia University, President Lee Bollinger announced Sunday evening that classes would be canceled Monday and Tuesday after a member of the community has been quarantined as a result of exposure to the coronavirus. (NYTNC)
8. Trump said he would request the Treasury Department to delay tax payments for individuals and businesses affected by the virus. He also repeated a pledge for payroll tax relief. He added that he would soon take “unprecedented” emergency action to provide financial relief to Americans affected by the virus. (NYTNC)

In these sentences, speech act reporting verbs, *announce, confirm*, and *add* are used to introduce the necessary measures in different areas taken by the government and the social organizations in response to the “unprecedented” emergency, and the new development in scientific research on the virus. This shows that the news reports in both countries are aware of using this type of reporting verbs to report related information, and to spread scientific knowledge, appropriate countermeasures and the precautionary measures to the public, which helps to achieve the purpose of popularization of science and the anti-epidemic work. Reporting the information of necessary response to the pandemic without delay in Chinese news media helps to strengthen international communication and promote international information sharing by telling what is being done in China.

The use of speech act reporting verbs shows that illocutionary force is given to reported information by the journalist. Thus, the reader’s interpretation of the reported information can be controlled or influenced by the journalist or the media (Yamashita, 1998; Xin and Gao, 2019). Each of these speech act verbs has a different kind of illocutionary force. For example, the use of “announce” indicates that the reported information is expressed in a public or official way, and even in a rather aggressive way, and the activities of the readers will therefore be influenced.

### 4.3. Mental reporting verbs

Mental reporting verbs, *believe* and *think*, only occur frequently in the American news corpus with 47 occurrences (13 occurrences per 10,000 words). The frequency of this type of reporting verbs is obviously lower than that of the other two types. In news reports, the use of these mental verbs indicates that the subject of the reported speech holds an uncertain view toward the current event and that the journalist (author) is uncertain of the trueness of the reported speech (Hyland, 2002). For example,

9. The same survey found that 12% of Democrats believe President Donald Trump is doing a good job handling the crisis and 23% believe Vice President Mike Pence is doing a somewhat or very good job. (NYTNC)
10. The Pew poll found that 77% of Republicans thought the media was exaggerating the risk, while 49% of Democrats shared that view. (NYTNC)
11. Many analysts believe that this moment, in the midst of the global coronavirus pandemic, represents perhaps the greatest opportunity these hackers have ever had to infiltrate the networks of American government agencies. (NYTNC)
12. During a press briefing Friday, Trump said he thought the restrictions in some states were “just too much,” repeatedly citing Virginia and the Second Amendment, and indicated that he did not think the gatherings would result in people spreading the virus. (NYTNC)

In the sentences above, mental reporting verbs *think/thought* and *believe* are used either to report the result of the public opinion survey or to introduce the speech by the authority which seems uncertain to the journalist or the press. In the former case, e.g., sentences (9) and (10), the introduced speech is concerned with people’s attitude toward the response of the government or the media to the crisis. This is closely related to the national conditions of the United States, the two-party system and the popular practice of public opinion survey, and shows common people’s involvement in and attitude toward the issue. In the latter case, the introduced speech is the subject’s personal attitude toward the possible consequence of or response to the pandemic, e.g., sentences (11, 12). The use of mental reporting verbs indicates that even the authority may be unsure of the consequences of the pandemic, and thus shows uncertainty toward the pandemic. By using the mental verbs frequently, the American news reports tend to present the interaction between the authority and the common people. The use of these mental verbs shows that the internal uncertainty of the subject of reported information is presented to the reader (Yamashita, 1998; Xin and Gao, 2019). Chinese news reports probably need to raise the awareness of using mental reporting verbs to express the opinions and attitude of the common people or the authority.

It is widely accepted that public opinion has significant influence on government policy making, even though the influence might be different in different countries. Public opinion survey organizations can influence the people’s understanding of the domestic and international politics, and these organizations with global influence almost all come from western countries, especially America (Zhai, 2014). It is possible that the government might achieve its political goal by fully utilizing the result of public opinion surveys. Public opinion survey organizations are deeply involved in the public discourse and are therefore closely related to the national discourse (Zhai, 2014). It seems that American media is likely to use the result of public opinion survey to help the government in decision making. This is probably caused by the different national conditions in China and United States.

### 4.4. Summary

In the “text” dimension, the three types of high-frequency reporting verbs and the linguistic realization of are identified and analyzed. The two media show both similarities and differences in the distribution of reporting verbs in news reports on the pandemic. Speech reporting verbs are most frequently used in both corpora, speech act reporting verbs occur more frequently in Chinese news reports, and mental reporting verbs occur frequently only in American news reports.

In the “discourse practice” dimension, the most frequent use of speech reporting verbs in both corpora shows the objective attitude and neutral stance of both media in the production of news texts. Chinese news tend to report with a comparatively higher degree of certainty toward the reported information since speech act reporting verbs occur more frequently in Chinese news texts. American news may report with uncertainty toward the reported information, as mental reporting verbs occur frequently only in American news texts. The difference in the degree of certainty shows difference in reporting strategy, which is controlled by the goal of the news reports determined by the authority and the need of the public.

In the “social practice” dimension, the difference in reporting strategy of the two media can be explained by different social context. At that time, the pandemic broke out suddenly in China with high infection rate, and is considered as a national public health emergency with greatest difficulty in prevention and control since the founding of the People’s Republic of China (Zheng and Zhao, 2020). The severe situation might cause panic among the public, and it was an urgent task for Chinese media to reveal the truth to the public and to the international community. Knowledge about the virus and countermeasures taken by the central government needs to be reported with higher certainty in order to relieve the tension and anxiety of the people. While the situation in America at that time was comparatively less serious, the reporting practice was conducted with comparatively lower degree of certainty, indicating uncertainty aspect of the pandemic; American news reports are likely to use the result of public opinion survey to help the government in decision making, probably due to the different national conditions in China and US. This shows that the power of the media is to shape governments and parties and that the power to represent things is largely a matter of how language is used (Fairclough, 1995).

## 5. Conclusion

### 5.1. Major findings

Using the corpus-based approach, this study examines the reporting practices in Chinese and American news report on COVID-19 pandemic from the perspectives of CDA. It is found that: (1) Chinese and American news reports tend to use roughly the same high-frequency reporting verbs in reporting the public health event. (2) The two corpora show difference in the distribution feature of high-frequency reporting verbs in terms of semantic category. Speech reporting verbs and speech act reporting verbs are frequently used in both corpora, while mental reporting verbs are frequently used only in the American news corpus; Chinese news reports probably need to raise the awareness of using mental reporting verbs to express the opinions and attitude of the common people or the authority. (3) Both Chinese and American news reports use speech reporting verbs most frequently, indicating comparatively objective attitude toward the current event, and can use speech reporting verbs and speech act reporting verbs to introduce the information with comparatively higher degree of certainty. American news reports frequently use mental reporting verbs to show the uncertainty aspect of the pandemic. This study indicates that different choice of reporting verbs shows difference in reporting strategy, and different reporting strategy might be caused by the different social and cultural context in China and United States.

### 5.2. Implications

The findings of this study can provide insights into the research on the practice of reporting strategies in news reports on emergencies in China for foreign audience. In face of the public health emergency, the responsibilities of Chinese news media include telling China’s story well, strengthening international communication, and promoting international information sharing (Zheng and Zhao, 2020). Emergencies like COVID-19 pandemic always have a huge impact on the society, and will become the hotspot of public concern in a very short time. And Chinese news reports therefore tend to report the information with higher degree of certainty rather than with lower degree of certainty, which will promote international information sharing.

The frequent use of mental reporting verbs in NYTNC indicates that American news media tend to use the public opinion surveys to show public concern or people’s attitude toward the current issue, which is probably related to the difference in national conditions, e.g., the two-party system in the country. The news report on emergencies in China for foreign audience has always been a big problem Chinese journalism (He, 2008), which will influence the construction of the national image and the international competitiveness of China in journalism. It is necessary for Chinese media to raise the awareness of using mental reporting verbs to express the opinions and attitude of the common people or the authority, indicating comparatively lower degree of certainty when dealing with public attitude in the event of the pandemic in the early stage.

### 5.3. Limitations and future research

The limitation of this study lies mainly in the comparatively small size of the corpora since the findings based on larger corpora of news reports on the same topic will be more convincing. The news data collected in this study are released in 2020, a little bit earlier, and the data made of recent news reports can better show the current situation reporting practices in news texts.

Future research on reporting practices in news reports can further the study of reporting verbs based on larger corpora made of latest news texts, or focus on the reporting manners or source of the reported speech in news texts from different news media.

## Data availability statement

The raw data supporting the conclusions of this article will be made available by the authors, without undue reservation.

## Author contributions

ZL performed the material preparation and data collection, and wrote the first draft of the manuscript. RZ and BL were responsible for the conception and design, interpretation of data, and critical revision of the manuscript. All authors contributed to the article and approved the submitted version.

## Conflict of interest

The authors declare that the research was conducted in the absence of any commercial or financial relationships that could be construed as a potential conflict of interest.

## Publisher’s note

All claims expressed in this article are solely those of the authors and do not necessarily represent those of their affiliated organizations, or those of the publisher, the editors and the reviewers. Any product that may be evaluated in this article, or claim that may be made by its manufacturer, is not guaranteed or endorsed by the publisher.
